# Analyzing atomic force microscopy images of virus-like particles by expectation-maximization

**DOI:** 10.1038/s41541-024-00871-7

**Published:** 2024-06-20

**Authors:** Rachel A. McCormick, Nicole M. Ralbovsky, William Gilbraith, Joseph P. Smith, Karl S. Booksh

**Affiliations:** 1 Department of Chemistry and Biochemistry, University of Delaware, Newark, DE 19716 USA; 2 Analytical Research & Development, MRL, Merck & Co., Inc, West Point, PA 19486 USA; 3 Process Research & Development, MRL, Merck & Co., Inc, West Point, PA 19486 USA

**Keywords:** Protein vaccines, Protein vaccines, Recombinant vaccine

## Abstract

Analysis of virus-like particles (VLPs) is an essential task in optimizing their implementation as vaccine antigens for virus-initiated diseases. Interrogating VLP collections for elasticity by probing with a rigid atomic force microscopy (AFM) tip is a potential method for determining VLP morphological changes. During VLP morphological change, it is not expected that all VLPs would be in the same state. This leads to the open question of whether VLPs may change in a continuous or stepwise fashion. For continuous change, the statistical distribution of observed VLP properties would be expected as a single distribution, while stepwise change would lead to a multimodal distribution of properties. This study presents the application of a Gaussian mixture model (GMM), fit by the Expectation-Maximization (EM) algorithm, to identify different states of VLP morphological change observed by AFM imaging.

## Introduction

Virus-like particles (VLPs) are self-assembled capsids formed of the proteins within the structure of a respective virus. VLPs are subunit conglomerations linked in a regular and repetitive fashion to form highly related, if not identical, overall structure to their parent virus^1,2^. Furthermore, VLPs are self-assembled in their host cell, and that same mechanism can be utilized ex vivo^1,3^. Therefore, VLPs produce an immune response in the human body capable of providing future protection from related viruses. Importantly, VLPs contain no DNA and therefore cannot reproduce or cause true infection, making them an excellent analog for the production of high immune response vaccines, as well as a significantly safer alternative for vaccine production compared to other methods^2,4^. VLPs can be key components of a drug substance, but are typically only an intermediate in the overall drug product. Further, many VLPs useful for vaccine production have been identified^2,5–8^, or are under investigation, and VLP-based vaccine production appears to present a fruitful option for the future of preventative vaccination science^9,10^.

The physical and morphological properties of any VLP is of paramount importance because their structure may directly control and facilitate their biological response. If the structure changes, the VLP may no longer faithfully induce antibodies to the parent virus and therefore the vaccine may potentially lose effectiveness^4^. Studying the physical characteristics of VLPs is required for better understanding the full effectiveness and production of VLP-based vaccinations. Of current interest is the human papillomavirus (HPV) vaccine based on the usage of HPV VLPs as antigens. HPV vaccines rely on the coverage from various genotypes, including types 6, 11, 16, 18, 31, 33, 45, 52, and 58, which in turn are assembled and stabilized by disulfide linkages of L1 proteins^11–13^. Specifically, temperature and pH play a large role in the stability of L1 disulfide linkages and how the VLP will self-assemble^11,14,15^. Further understanding of the VLP morphological changing processes can provide needed insight for the advancement and improvement of VLP manufacturing and VLP-based vaccine production.

Common spectroscopic techniques are spatially limited to the diffraction limit of the excitation wavelength^16^, making individual study of the ~50 nanometer-sized HPV VLPs impractical by confocal microscopy. Several imaging methods exist that can be used for analysis of VLPs, including scanning electron microscopy (SEM)^17^, transmission electron microscopy (TEM)^18,19^, and cryo-electron microscopy (cryo-EM)^20,21^. While electron microscopy methods have some unique advantages, atomic force microscopy (AFM) allows for high spatial resolution, direct observation of individual particles, rapid analysis with no sample preparation, low analysis cost, and potential combination with other molecular-probing tools (i.e., AFM with spectroscopic techniques). AFM can be used to not only probe the size and morphology of the VLPs^22,23^, but also their elasticity^15,24^. Since the properties of the viral capsid are tied to the structure, monitoring morphology or elasticity changes can give insight into molecular interactions. Easily identifiable structural changes in the VLPs may potentially indicate deterioration^25,26^. AFM can be used for nano-indentations of the capsid surface, which may help elucidate further information of capsid stability, formation mechanism, and viral uncoating^24,27^. Nano-scale modulations of the viral capsid may also be used as a predictor for VLP application success before a point of failure^24^. However, it is important to note a relatively stiff AFM tip can be used to compress the target VLPs to the point of structural failure in efforts to probe internal changes of the particle^27^.

Understanding the physical and chemical properties of VLPs is important for maintaining the effectiveness of vaccinations, where VLPs can function as a key intermediate in the drug substance. Marchetti et al. shows that AFM can be used to probe VLP structural integrity via a nano-indentation process to understand the force required to destabilize a single virion^24^. Using this information, small mutations of the VLP proteins were shown to cause up to a threefold increase of capsid strength^24^. Similarly, a relatively stiff AFM tip can be used to compress the target VLPs to the point of structural failure in efforts to probe internal changes of the particle^27^. Sharma et al. shows that Spytag-S SARS-CoV-2 VLPs exhibit structural instability with short exposures to mildly elevated temperatures^15^. Gonzalez-Dominquez et al. uses multi-frequency AFM to characterize HIC-1 Gag VLPs^28^. We propose simple AFM imaging can detect and exemplify conformational changes in VLP structure, and therefore VLP effectiveness, before full particle deterioration. With the aid of Bayesian statistical interpretation, further information can be elucidated from AFM imaging without the use of expensive electron microscopy instrumentation.

Subsequent to collection of AFM images, a variety of analysis strategies can be employed for extraction of useful information embedded within the images. Typically, analysis of AFM images centers on determination of particle size information – that is, individual particles are identified and analyzed for their size. Various plots, such as histograms or box-and-whisker visualizations, are generated to indicate size distributions from the image^29^. The limit of traditional statistical analysis applied to AFM images is frequently encountered when analyzing heterogeneous mixtures, as compared to that of homogenous samples. Moreover, when observed samples belong to a homogeneous population, the distribution of sample properties can be adequately modeled by one set of parameters that define the distribution. In many cases, a Gaussian distribution fits the population to be studied and hence, the ensemble of collected data can be accurately summarized by the mean and standard deviation of the data. However, in some cases, an observed sample or system may consist of multiple groups—each group with a different set of descriptive parameters. Even if each group in heterogeneous mixtures is Gaussian in character, these groups may not be well described by a simple population mean and standard deviation analysis. Moreover, there is a greater level of nuance in the data (e.g. the mean, standard deviation, and proportion of each group) that must be extracted to fully characterize and understand the observed collection of data. A Gaussian Mixture Model (GMM) may more accurately describe this type of data^30^. However, accurately determining the descriptive parameters of a GMM is not straightforward.

The parameters of a multi-factor GMM cannot be linearized, and therefore these parameters cannot be directly estimated by a linear least squares approach; instead, non-linear least squares algorithms, such as Levenberg-Marquardt^31,32^ or Trust Region^33,34^, are often enlisted. However, the large number of parameters to be fit, coupled with the flexibility of non-linear least squares algorithms, leads to both of these procedures readily becoming trapped in non-optimal local minima. Consequently, a non-linear least squares approach works best when GMM means and standard deviations are well known and only the mixing parameters are uncertain; this is the case with applications that include X-ray photoelectron spectroscopy (XPS)^35,36^. Some software packages avoid acknowledging the problem of local-minima traps by implementing an iterative curve-fitting procedure based on user assessment of model quality; such procedures lack statistical rigor and reproducibility. A robust alternative to non-linear least squares methods for fitting a GMM is estimating the latent parameters through a Maximum Likelihood (ML) approach. ML methods optimize the likelihood of a set of parameters being correct, given a collection of observed data.

To the best of our knowledge, GMM has not yet been reported to study the bulk morphology of nanoparticles imaged by AFM in general, much less the morphological changes of VLPs. The most similar application of GMM has been the employment of GMM to better understand cell surface architecture using single molecule force spectroscopy data^37,38^. GMM have also been combined with computational chemistry simulations to estimate the most-probable conformations of large proteins^39,40^. In a related effort, a 3D GMM was developed to create atomic-level density maps of functional groups from cryo-electron microscopy images^41^. As such, this is the *first* report of GMM being applied for understanding and investigating AFM images.

Consequently, this work presents two advances to analytical methodology. First, the collected data explores the potential of nano-indentation AFM to better understand VLPs by indirectly probing internal structural integrity as opposed to topography^24,42^. Second, to fully analyze the AFM data, GMM is applied for the *first* time to categorize morphology of VLPs. Taken together, the results can help elucidate key phenomena surrounding VLP properties.

## Results and discussion

### Analyses of VLP images by area

Classical statistics presents an inconclusive trend for mean VLP area for the compressed VLPs as the particles age over the first 4 h in solution at 20 °C. The mean VLP area increases from 1509 nm^2^ to 1668 nm^2^ over hours 2 to 3. However, the mean observed VLP area decreases to 1397 nm^2^ after 4 h of thermal aging. Across the same period, the standard deviation of the observed VLP distribution increases from 183 nm^2^ to 248 nm^2^ to 307 nm^2^.

**Fig. 1:**
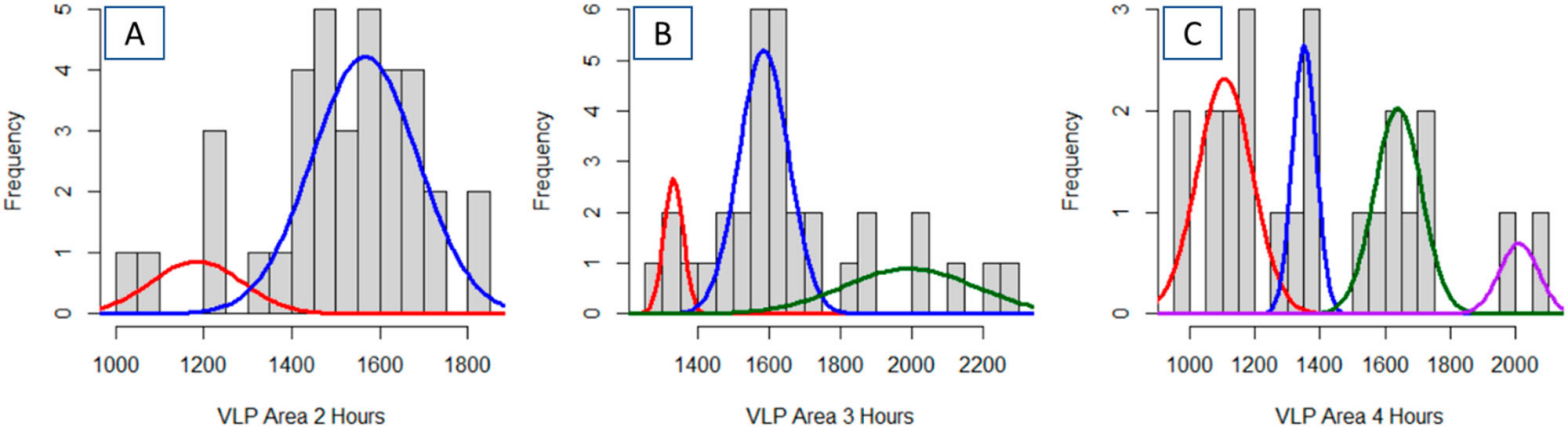
The gaussian mixture model shows an increasing spread in particle size distributions as the VLPs thermally age. Optimized Gaussian Mixture Model profiles for the single pass AFM images based on VLP area following 2-hours (**A**), 3-hours (**B**) and 4-hours (**C**) of thermal aging. The VLP suspensions were left at room temperature for 2–4 hours prior to placement on a mica surface.

Histograms of three aged VLP collections show that their distributions are non- normal (Fig. 1) and consequently, the sample mean and standard deviation do not present a complete or nuanced view of VLP morphological changes. Cursory study of the histograms shows that the distributions become skewed towards larger VLP areas. All three samples have a significant number of VLPs around 1600 nm^2^, but later aging times include a selection of VLPs of approximately 2000 nm^2^.

**Fig. 2:**
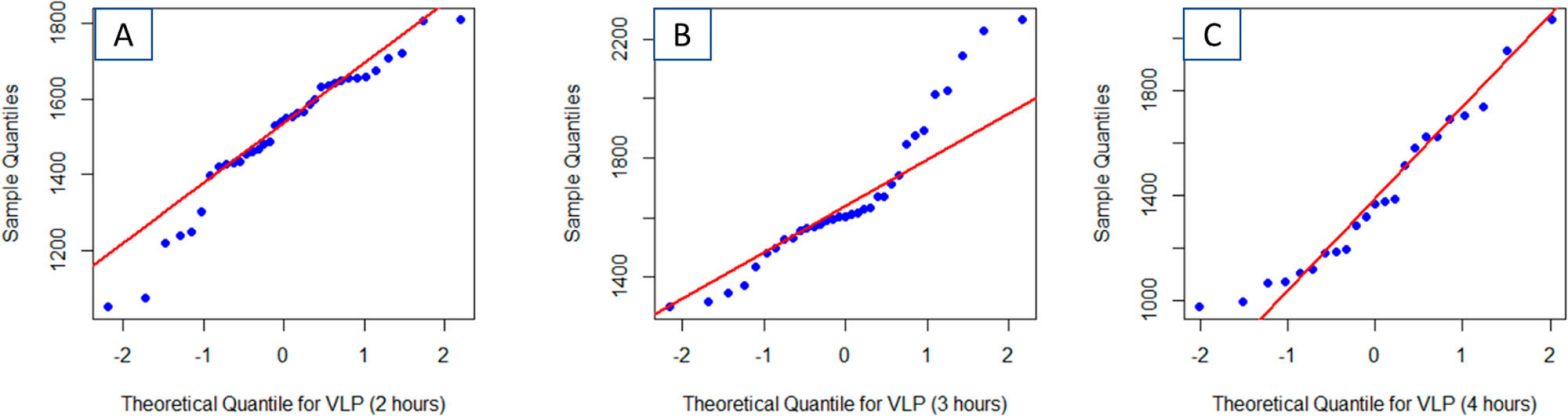
Quantile–quantile plots of VLP area extracted from the AFM images verses a theoretical Gaussian distribution show that the thermally aged particles are not normally distributed. Plots are shown for samples following 2 h (**A**), 3 h (**B**) and 4 h (**C**) of thermal aging. That the Q–Q plots are not linear indicates significant deviation from a single Gaussian accurately describing the VLP distribution. The included red line represents the perfect, noiseless, data, and model.

Quantile-Quantile (QQ) plots better illuminate the deviation from normality of the three VLP populations (Fig. 2). A QQ plot is a graphical technique for determining if a set of observed data belongs to a particular theoretical distribution. A quantile is the fraction of observations below a given value. For an ordered (i.e., lowest value to highest value) data set, the quantiles of the observed data are plotted against the quantiles of the theoretical distribution. Here, the theoretical quantiles are normalized to be standard deviations from the mean because the data is assumed to be normally distributed. Were the observed data to adhere to the theoretical distribution, all points would align along the reference line (Fig. 2, red). All three collections of VLP particle sizes show significant deviations from linearity, indicating that none are normally distributed.

If the collections of VLPs exist as a mixture of distinct normal distributions, each with a different mean area and standard deviation, this data can be modeled by a GMM and deconvolved by the EM algorithm. Each set of VLPs extracted from collections of three AFM images was modeled with 2, 3, 4, and 5 latent Gaussian distributions. The EM algorithm extracts the mean, standard deviation, and relative contribution of each component to the mixture. The optimal complexity of a GMM for each collection was determined based on the fit of each model, as expressed by the log likelihood value and the linearity of the QQ plots for each model. When the inclusion of an additional Gaussian no longer significantly improves the log likelihood and linearity of the QQ plot, the GMM is assumed to be optimal. The final GMM can be expressed in tabular form (Table 1) or graphical form (Fig. 1).

**Table 1:** Fitted Gaussian Mixture Models parameters for thermally aged VLPs imaged by a single pass of an AFM tip

Description	Number of VLPs, total	Fraction	Mean	Sigma	95% C.I. of mean
2 hours	36	0.15	1184	103	51
		0.85	1566	120	60
3 hours	33	0.12	1331	27	18
		0.60	1585	69	46
		0.28	1993	185	120
4 hours	23	0.41	1107	85	95
		0.20	1351	35	39
		0.30	1639	71	80
		0.09	2011	59	66
All VLPs – 3 factors	92	0.66	1450	241	87
		0.27	1613	56	20
		0.07	2093	128	46
All VLPs – 4 factors	92	0.07	1054	48	20
		0.59	1479	194	81
		0.25	1617	54	22
		0.09	2045	150	62

Individual VLP area is the extracted descriptor from each image. Data for each aging time is determined from the ensemble of three AFM images.

**Fig. 3:**
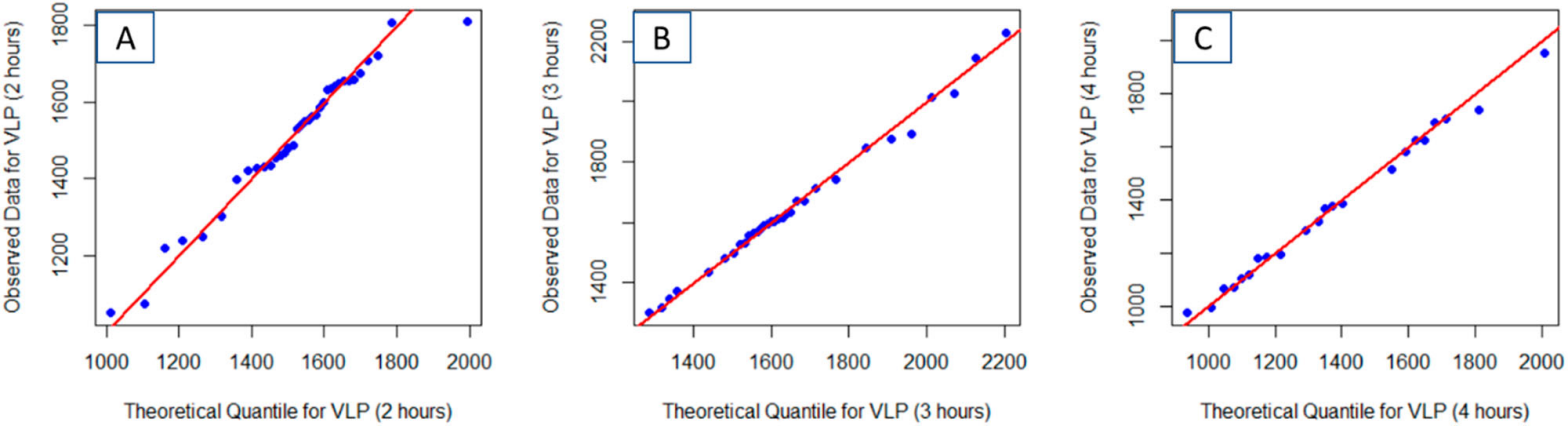
Quantile–quantile plots of VLP area extracted from the AFM images verses the optimized Gaussian Mixture Model distribution show that the thermally aged particle distribution conforms to the GMM. Plots are shown for samples following 2 h (**A**), 3 h (**B**) and 4 h (**C**) of thermal aging. That the Q–Q plots are linear indicates confidence that the GMM accurately describe the VLP distribution. The simplest GMM that yielded a linear Q–Q plot was retained. The included red line represents the perfect, noiseless, data and model.

Application of the EM algorithm (Table 1 and Fig. 3A) indicates that after two hours of thermal aging, the VLPs are best modeled by a multi-normal distribution with mean areas of 1184 nm^2^ (15% of the VLPs) and 1556 nm^2^ (85% of the VLPs). After 3 h, three intrinsic normal distributions are extracted from the GMM (Table 1 and Fig. 3B): 1331 nm^2^ (12% of the VLPs), 1585 nm^2^ (60% of the VLPs), and 1993 nm^2^ (28% of the VLPs). After 4 hours of aging, four components were observed (Table 1 and Fig. 3C): 1107 nm^2^ (41% of the VLPs), 1351 nm^2^ (20% of the VLPs), 1639 nm^2^ (30% of the VLPs), and 2011 nm^2^ (8% of the VLPs). For the 4-h images, four Gaussians were chosen based on the visual fit of the model to the data (in Fig. 3C). Cursory comparison of the QQ plots for the GMM (Fig. 3) indicates significantly better fit of the model to the data than with a single normal distribution (Fig. 2). Pearson’s Chi-squared test for count data indicated that the modeled distributions at three separate times are statistically different (*p* < 0.01).

The 95% confidence interval for the mean value of each intrinsic distribution can be estimated by $\pm t\sigma /\sqrt{n},$ where *t* is the tabulated Student t-value for *n*-1 degrees of freedom, σ is standard deviation of the fitted normal distribution, and *n* is the number of observed samples in the distribution (Table 1, column 5). For each Gaussian in the GMM, determination of *n* is not straight forward. Here *n* for each distribution is estimated as the number of observations divided by the number of distributions in the GMM. Hence, *n* is 18, 11, and 6 for these three collections of VLPs analyzed by AFM.

Considering the three GMM analyses as a whole, three trends are evident. All optimized GMMs extract a set of VLPs with an area of approximately 1600 nm^2^ and are of comparable size based on the 95% confidence limits. The two longer aging times exhibit VLPs with a comparable average area around 2000 nm^2^. These larger observed structures would be expected because as the bonds within the VLPs may change, the VLPs may become structurally less rigid and consequently flatten to a larger area^15,43^. The 2 h and 4 h VLPs present a smaller structure with an average area around 1100 nm^2^, while the 3 h and 4 h VLPs have a larger structure around 1300 nm^2^. Given the small number of AFM sampling sites and number of VLPs analyzed, these two distributions might converge to a single normal distribution in a larger collection of AFM images of VLPs.

**Fig. 4:**
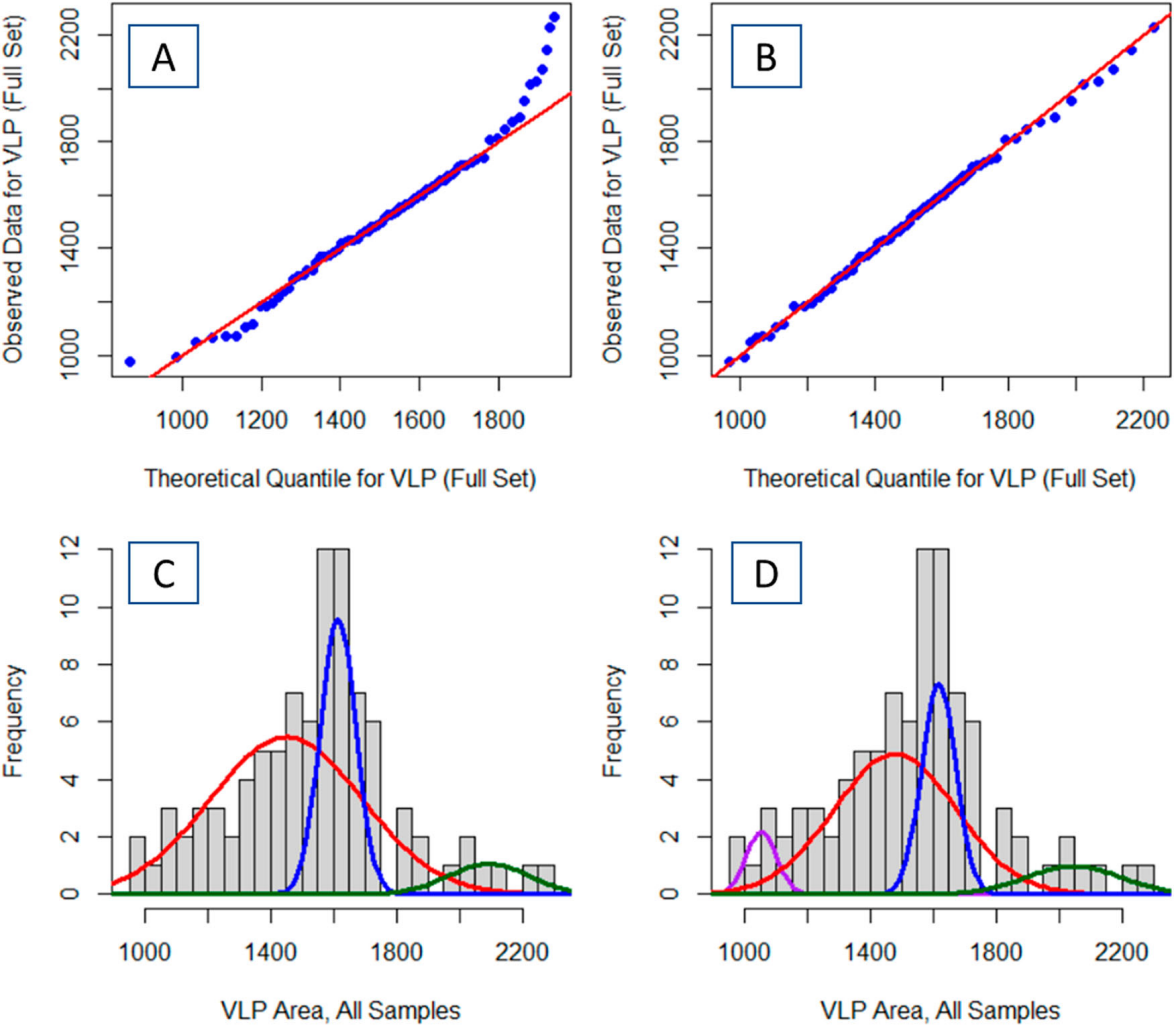
The four-component Gaussian Mixture Model fits the VLP size distribution better than the three-component Gaussian Mixture Model. The improvement of fit in increasing from a three-component Gaussian Mixture Model (**A**, **C**) to a four-component Gaussian Mixture Model (**B**, **D**) is evident in the increased linearity of the respective quantile–quantile plots (**A**, **B**).

Analyses of all 92 determined VLP areas from the three aging times as a single distribution resolves four distinct groups of VLP (Fig. 4D). While a three factor GMM model appears to be a reasonable description of the VLP histogram (Fig. 4C), the QQ-plot of the model shows significant deviations at the large area end of the particle distribution and minor deviations at the small area end of the VLP distribution (Fig. 4A). Adding a fourth term to the GMM provides a much better fit of the model to the data, reducing deviations on both extremes **(**Fig. 4B). The parameters of the 3 and 4 factor GMM are similar—the ~1615 nm^2^ centered distribution only differs by a mean of 4 nm^2^, standard deviation of 2 nm^2^, and a 2% contribution between the models. However, inclusion of a fourth component in the model adds a factor explicitly modeling the distribution of the smallest particles (µ = 1054 nm^2^; σ = 48 nm^2^) and slightly shifts the estimated means and standard deviations of the other two distributions (Table 1). Consequently, the net observed distribution of VLP sizes are better described by a 4-factor GMM model than with a 3-factor GMM model. Pearson’s Chi-squared test for count data indicated that the 3-factor and 4-factor models are statistically different at *p* = 0.051.

Applying the GMM to analyses of all 92 VLPs provides similar results to that of analyzing the three aging processes separately. The ensemble set of VLPs returns distributions centered at 1054 nm^2^, 1479 nm^2^, 1617 nm^2^, and 2045 nm^2^. Every distribution of VLPs resolved by applying a GMM to individual aging times aligns with the four distributions observed with analyses of the ensemble data. The 3-h thermal aging 1993 ± 120 nm^2^ and 4-h thermal aging 2011 ± 66 nm^2^ centered distributions are statistically indistinguishable from the ensemble 2045 ± 62 nm^2^ centered distribution. Similarly, the ensemble 1617 ± 22 nm^2^ are matched with the 2-h 1566 ± 60 nm^2^, 3-h 1585 ± 46 nm^2^, and 4-h 1639 ± 80 nm^2^ centered distributions. However, there is more deviance in the resolved GMM parameters between the ensemble and individual aging times at the low area end of the sample set. With the individual aging times, the mean value of the resolved distributions with the smaller areas all lay between the 1054 nm^2^ and 1479 nm^2^ centered distributions. This issue could be explained by the low number of VLPs that inherently belong to the smallest distribution. For example, for 2-h, the 1184 nm^2^ centered distribution contains only 15% of the parent distribution (i.e., 5 or 6 VLPs total). It is unsurprising that the GMM would fail to resolve 2 sub-populations and estimate the mean to be between the 1054 nm^2^ and 1479 nm^2^ centered distributions.

### Analyses of VLP images by VLP width (single AFM tap)

Manually determining the width of the VLP in each image enabled observation of more VLPs in comparison to employing the area-based method discussed previously. With the width-based method, 54, 33, and 42 individual particles were extracted from the three thermal aging times compared to 36, 33, and 23 VLPs by the area-based method. It is appropriate to note in the following section where each VLP was scanned twice by AFM prior to determining the VLP area and width, less than 10 VLP areas could be reliably determined across three times, yet 98 individual VLP widths were calculated.

**Fig. 5:**
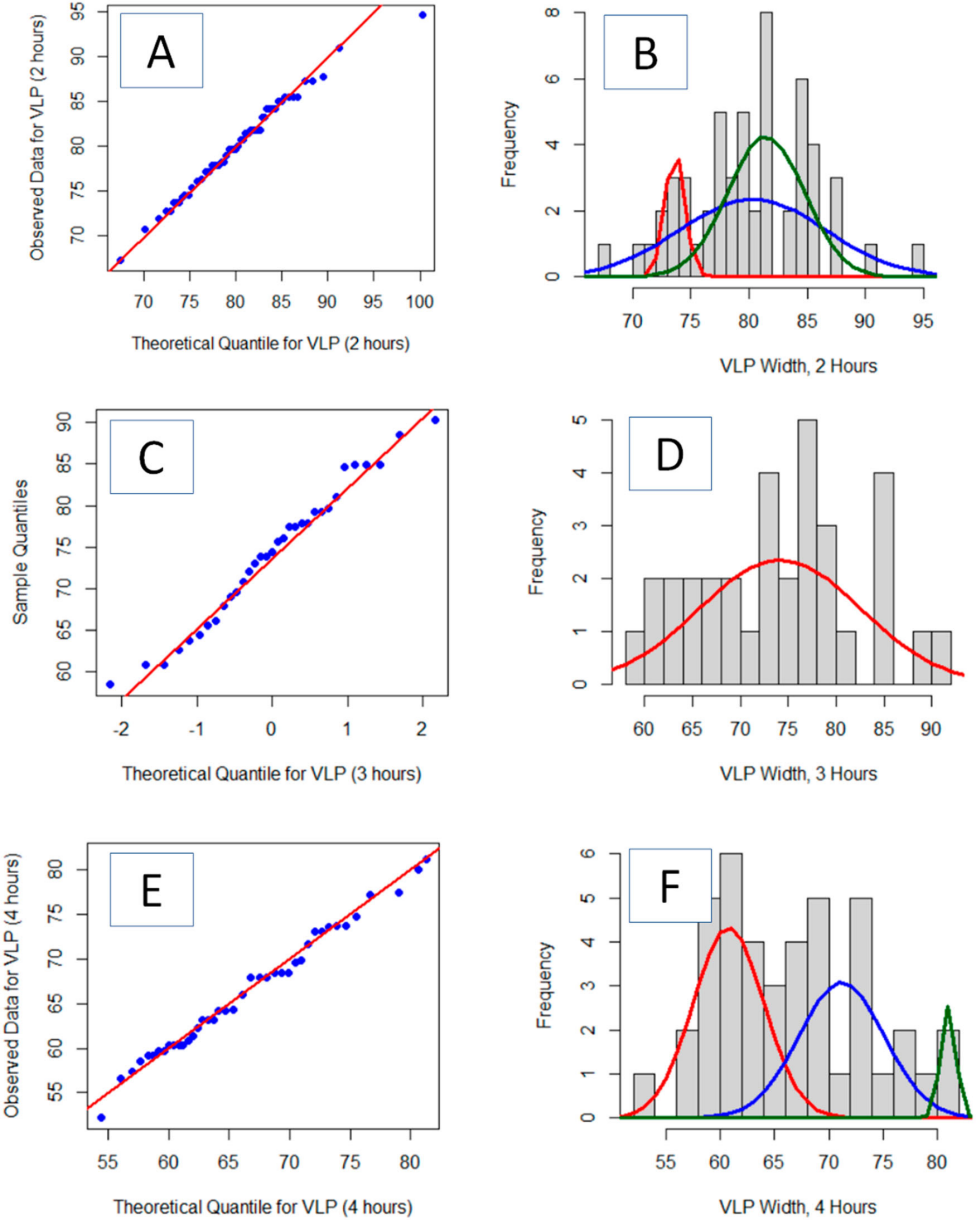
The optimized Gaussian Mixture Model shows a good fit to the distribution of VLP widths. Quantile–quantile plots and optimized Gaussian Mixture Model fit to the VLP widths extracted from the AFM images following 2 h (**A**, **B**), 3 h (**C**, **D**) and 4 h (**E**, **F**) of aging following a single nanoindentation AFM pass. That the Q–Q plots are linear indicates confidence that the GMM accurately describe the VLP distribution. The most simple GMM that yielded a linear Q–Q plot was retained. The included red line represents the perfect, noiseless, data and model.

Analyses of the width-based collection of VLP data presents a slightly less complex view of the VLP populations at each time as compared to the area-based analyses of the VLP (Table 2 vs Table 1). Perusal of the QQ-plots shows good linearity for each model (Fig. 5A, C, E). For the 2-hour time, both methods present the majority of the VLPs being larger, with a smaller population (15% vs 10%) being of lesser dimension (Fig. 1A vs. Fig. 5B). The width-based measurements model the larger VLPs with 2 factors; however, the mean values of both distributions are statistically indistinguishable. For the 3-h time, the model fit for the width-based measurements did not improve when using more than one normal distribution. This is in contrast to the area-based analyses that was optimally modeled with 3 normal distributions—each with a statistically different average area (Fig. 1B vs. Fig. 5D). Similarly, the area-based analyses for the 4-hour time identifies 4 unique VLP distributions, each with a statistically different mean area while the width-based analyses only identified 3 unique populations (Fig. 1C vs. Fig. 5F). Pearson’s Chi-squared test for count data indicated that the modeled distributions at three separate times are statistically different (*p* < 0.01).

**Table 2:** Fitted Gaussian Mixture Model parameters for thermally aged VLPs imaged by a single pass of an AFM tip

Description	Number of VLPs, total	Fraction	Mean	Sigma	95% C.I. of mean
2 h	54	0.10	73.6	0.83	0.4
		0.46	80.3	6.22	3.2
		0.44	81.4	3.28	1.7
3 h	33	1.00	74.1	8.47	2.9
4 h	42	0.50	60.8	3.24	1.9
		0.43	71.2	3.92	2.3
		0.07	81.0	0.70	0.4
All VLPs – 2 factors	129	0.23	61.8	3.60	0.6
		0.77	78.0	6.25	1.1

Individual VLP width is the extracted descriptor from each image. Data for each aging time is determined from the ensemble of three AFM images.

**Fig. 6:**
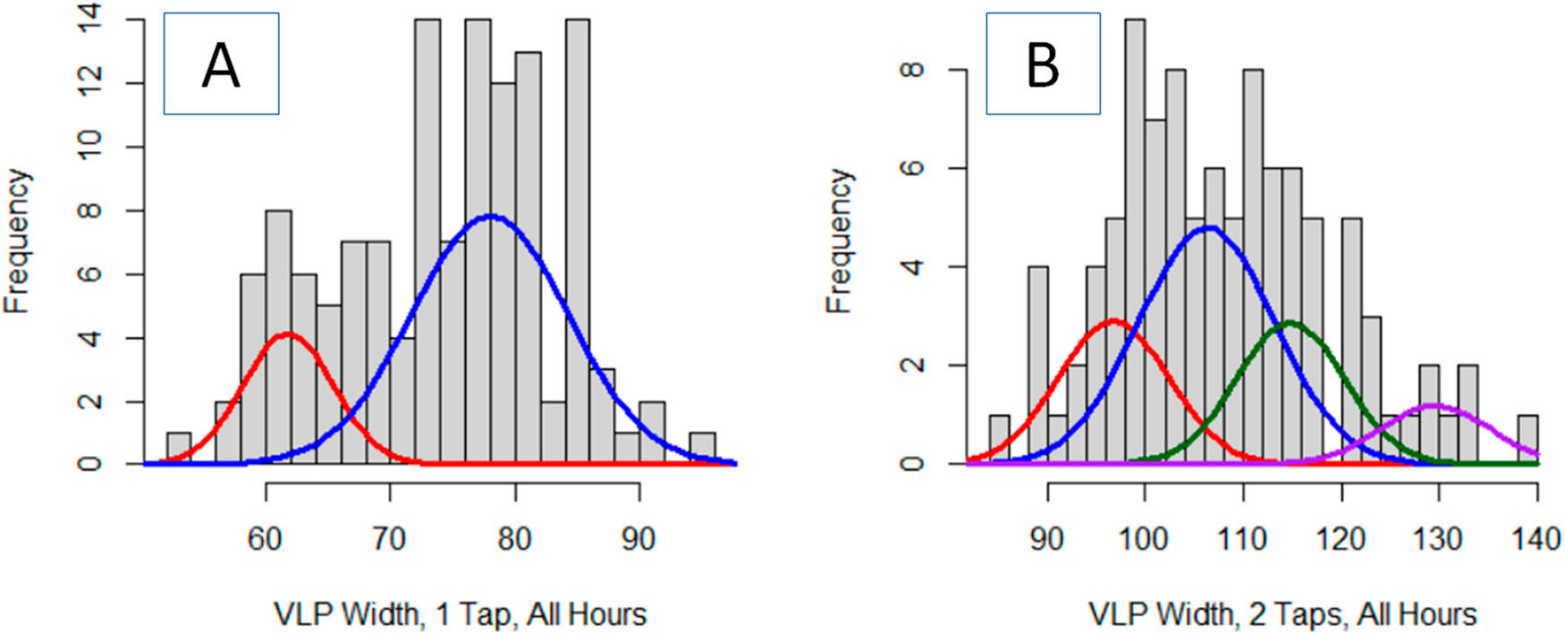
Comparison of optimized Gaussian Mixture Models for single and double pass AFM imaging indicate that the VLP widths are significantly wider after the second pass with nanoindentation. Distributions presented are from the ensemble all AFM images collected with a single AFM pass (**A**) and a double AFM pass (**B**).

The differential in model complexity between analyses of AFM images by area-based and width-based approaches for the VLPs holds when all nine collected images, across three aging times, are combined into a single population (Fig. 4D vs. Fig. 6A). The area-based analysis resolved 4 distributions of VLPs—two minor components that were either smaller (7%) or larger (9%) than the two main factors that constituted 84% of the particles (Table 1). However, the width-based analysis only resolved two main components, seemingly unable to extract the smaller and larger minor components. Of course, without further analyses of a larger data set, it is impossible to conclude with certainty whether the 2-component or 4-component model more is the more faithful description of the true VLP distribution.

### Analyses of VLP images by VLP width (double AFM tap)

Because the protocol for estimating VLP area mostly fails when individual particles form clusters or are touching, an alternate procedure to collect AFM images was investigated. Here a rapid, low spatial resolution AFM image of a large area was collected to identify regions of interest with the greatest number of non-contiguous VLPs. Those regions of interest were then resampled at higher spatial resolution. As such, the VLPs were double sampled during analysis. Unfortunately, with the stiff AFM probes, this resulted in wider (80–140 nm vs. 50–100 nm) and flatter VLPs observed during analyses. Ultimately, the resampled VLPs were too flat at the edges to determine the VLP boundary for the area-based procedure. Consequently, only width-based models could be constructed.

**Fig. 7:**
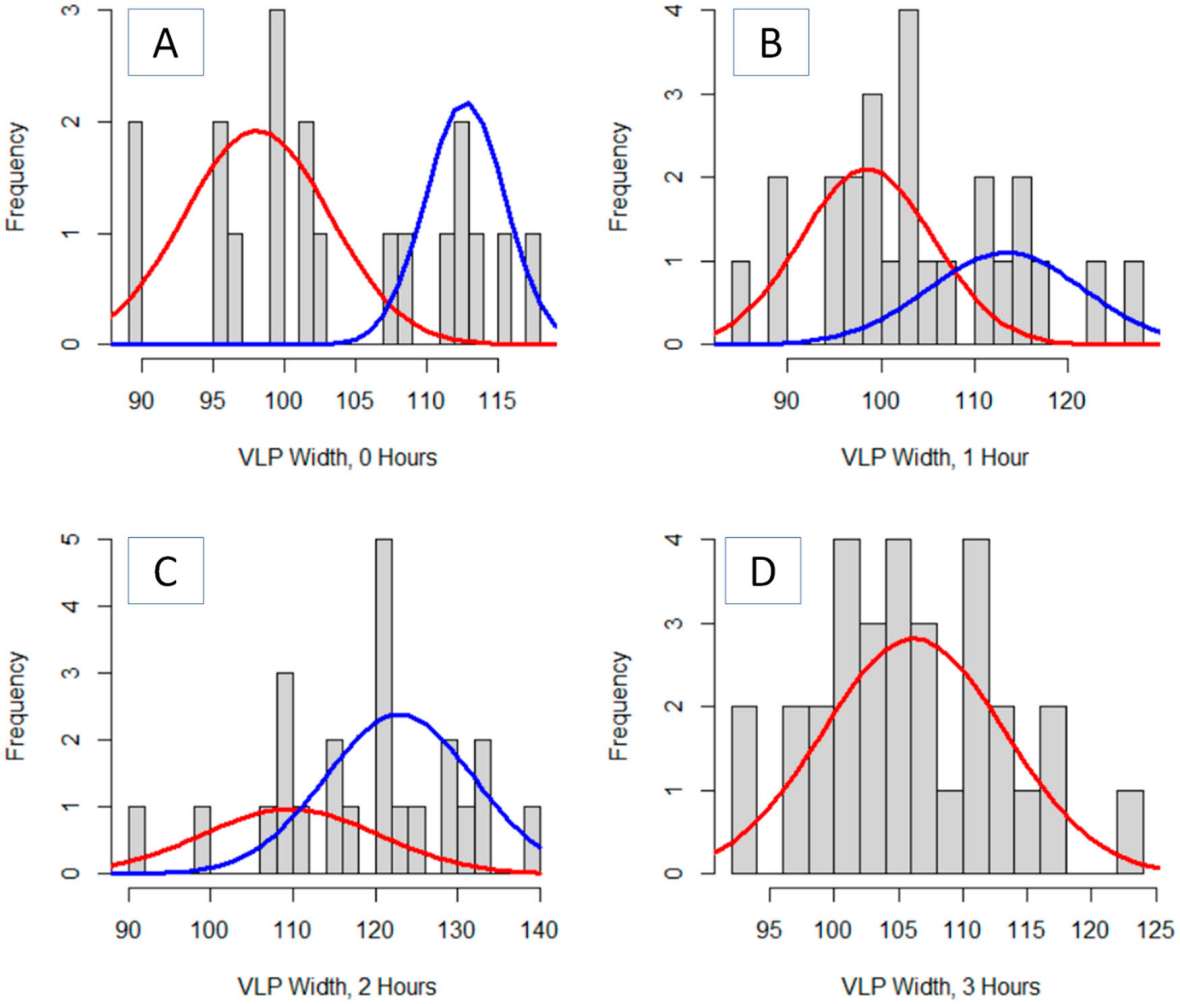
Optimized Gaussian Mixture Model fits to the VLP widths extracted from the double pass AFM imaging show a one or two component model. Distributions presented are AFM images following 0 h (**A**), 1 h (**B**), 2 h (**C**), and 3 h (**D**) aging following a second nanoindentation AFM pass.

For the 0-, 1-, and 2-h times, EM analyses indicated a bi-normal distribution (Fig. 7). With 0-h and 1-h, the mean of the two distributions (the first at ~98 nm and the second at ~113 nm) are statistically indistinguishable at the 95% confidence interval (Table 3). However, after 2-h, both distributions have a statistically greater mean. The smaller of the two distribution means increases from ~98 nm to 110 nm and the larger of the two distribution means increases from ~113 nm to 125 nm. This may be potentially indicative of the VLP losing rigidity during aging and spreading out over a larger area following AFM compression^15,43^. By comparison, the 3-h data was optimally modeled with a single Gaussian distribution. This distribution lacked features with a width greater than the 130 nm present in the 2-h data. One possible explanation may be that further aged VLPs were tapped sufficiently flat to not rise above the baseline noise of the image and image processing.

**Table 3:** Fitted Gaussian Mixture Models parameters for thermally aged VLPs imaged by a single pass of an AFM tip

Description	Number of VLPs, total	Fraction	Mean	Sigma	95% C.I. of mean
0 h	19	0.62	98.1	5.11	3.7
		0.38	112.7	2.81	2.0
1 h	25	0.62	98.5	7.05	4.3
		0.38	113.3	8.27	5.0
2 h	23	0.33	109.9	10.8	6.9
		0.67	123.0	8.98	5.7
3 h	31	1.00	106.2	7.08	2.5
All 4 h	98	0.23	96.8	5.6	2.3
		0.46	106.3	6.9	2.8
		0.22	114.8	5.5	2.3
		0.09	129.6	5.6	2.3

Individual VLP width is the extracted descriptor from each image. Data for each aging time is determined from the ensemble of three AFM images.

Analyzing the ensemble data from all 12 AFM images, spanning 4 aging times, indicates four normal sub-populations of VLP diameters (Table 3). However, the symmetric spacing and widths of the three leftmost Gaussians (Fig. 7B) are consistent with describing a platykurtic distribution by normal curves. Without a more extensive investigation, it is not feasible to determine whether the double tapping of the AFM analyses leads to a wider, non-normal distribution of VLP widths, or if this one set of data was anomalously wider. However, given that the EM algorithm did not model the data by fitting two Gaussians centered at the 100 nm and 110 nm spikes in the histogram lends credence that this is a single platykurtic, not bi-modal Gaussian distribution of VLPs. Consequently, the ensemble data is better viewed as two distributions of VLP widths across all observed aging times.

The totality of our novel methodology – using AFM in conjugation with GMM fit by the EM algorithm – provides the unique opportunity to investigate the bulk morphology of VLPs and the potential to identify VLP morphological changes. Herein, we report VLP morphological changes are occurring due to observances in the shape and diameter of the VLPs. The cause of these changes in VLP shape and size may be due to a multitude of factors, including room temperature aging, local temperature and pH alterations, stabilization buffer contents, and number of freeze-thaw cycles^15,43,44^. Moreover, the purified HPV VLP intermediates studied herein were specifically selected for straightforward analytical method development, in which case these VLP intermediates allows for a sample of only particles to be investigated (i.e., no drug product formulation components). The definitive causation of any VLP shape or size changes would indeed require further studies. Notably, the methodology showcased here illustrates, for the *first* time, the potential of nano-indentation AFM in combination with GMM and EM to probe the internal VLP structural integrity, as opposed to topography of VLPs, to reveal information about changes in VLPs.

## Methods

### Preparation of virus-like particles (VLPs)

Human papillomavirus (HPV) type 11 virus-like particles (VLPs) were obtained from Merck & Co., Inc., West Point, PA, USA. The VLPs were prepared following a similar manner to standard protocols^14,45,46^. Briefly, HPV type 11 L1 protein are expressed and self-assembled into a VLP structure via a recombinant *Saccharomyces cerevisiae* (yeast) expression system containing the genome coding for the HPV type 11 L1 protein. The self-assembled VLPs were subsequently purified. In order to study a representative sample of only particles for straightforward methodology development, purified HPV VLP intermediates were analyzed herein, in which these drug substance intermediate particles did not undergo any vaccine drug product formulation processes. HPV type 11 VLPs were also purchased from Creative Diagnostics (Shirley, NY, USA) for additional analysis.

### Collection of atomic force microscopy (AFM) images

VLP samples were thawed and diluted 20X in buffer (0.5 M NaCl, 0.012% Tween 80, 20 mM Histidine, pH 6.2). 10 µL were deposited on freshly cleaved mica disks (V1 grade, Ted Pella, Inc.) and allowed to incubate for 10 s before being rinsed with DI water 10 times in 10 µL aliquots. VLPs were aged at room temperature in buffer and sampled immediately after thawing as well as at 30 min time intervals for AFM imaging. An AIST-NT OmegascopeTM 1000 AFM (Horiba) in tapping mode was used to collect 1 µm × 1 µm images. All AFM imaging was carried out under ambient conditions in air. Topography imaging was performed using RTESPA-300 cantilevers (Bruker) with a mean spring constant of k = 40 N/m and tip radius of 8 nm. A setpoint amplitude of 20 nm and scan rate of 1 Hz was used.

### Analyses of AFM images

**Fig. 8:**
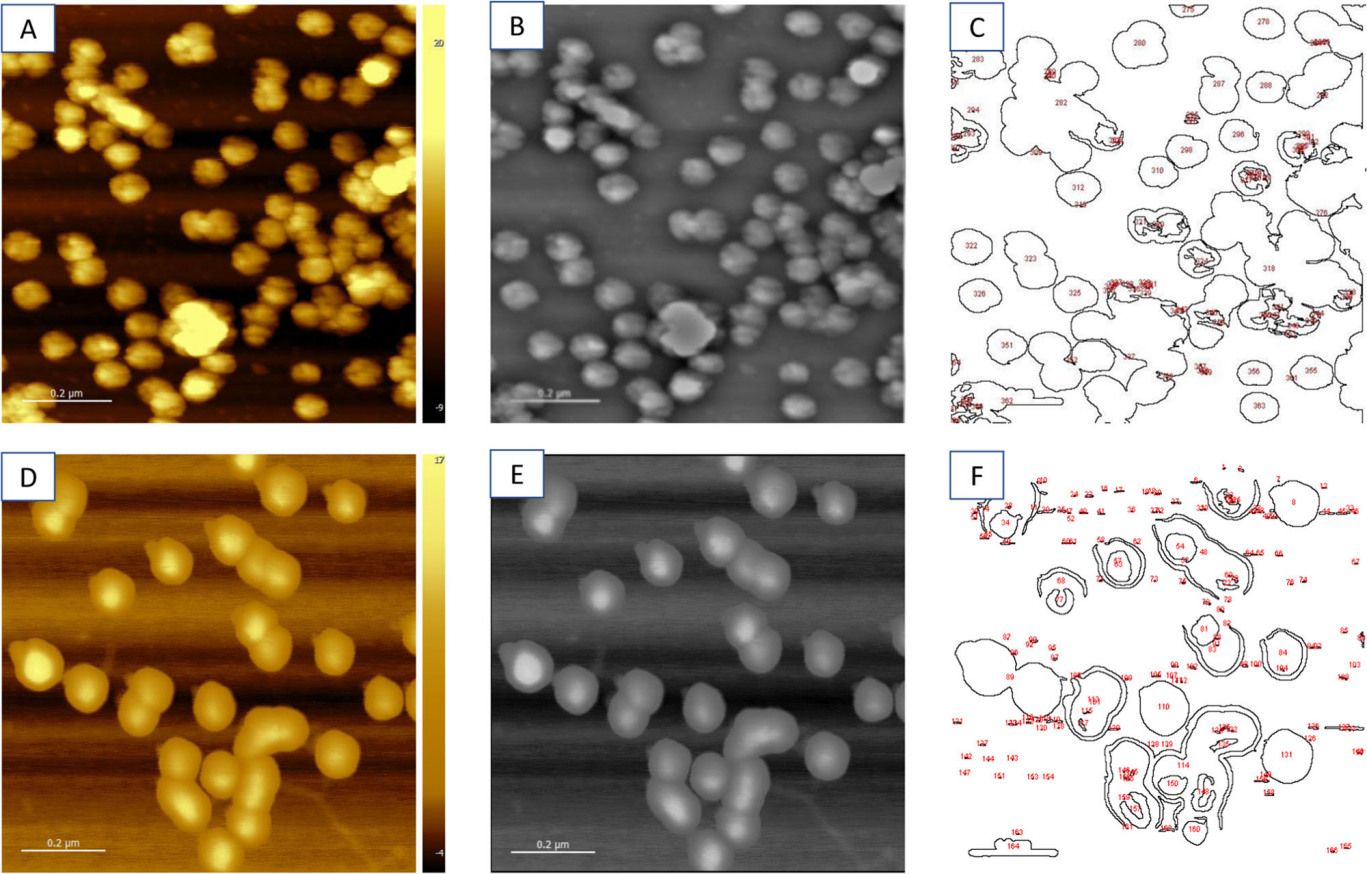
Analysis of the AFM images enables extraction of estimated particle sizes. Processing of AFM images to determine VLP area for the single passed (**A**–**C**) and double passed (**D**–**F**). The raw AFM images (**A** and **D**) were converted to 32-bit grey scale and the edges were enhanced by applying a 2D FFT filter in ImageJ (**B** and **E**). After manually adjusting the threshold to maximize the number of fully resolved VLP, the ‘Find Edges’ routine was applied to create a VLP edge map (**C** and **F**). Individual VLP area was determined by ImageJ integrating within each numbered contour.

All AFM images were saved as .jpeg images and processed in the ImageJ^47^ wrapper FIJI Is Just ImageJ (FIJI)^48^. The .jpeg images (Fig. 8A, D) were converted to 32 bit gray scale and smoothed with a Fast Fourier Transform (FFT) bandpass filter using default settings (Fig. 8B, E). Additional image conditioning tools in FIJI to enhance the contrast and sharpen the images were investigated and did not substantially improve the analyses. To determine the width of each VLP, the widest lateral point (x axis) was calculated manually in pixels and converted to nanometers using the scale bar provided by the AFM software. The scale ranged from 103 pixels per 200 nm to 125 pixels per 200 nm across all images.

Further processing was required to determine the area of each isolated VLP. First, the ImageJ ‘Find Edges’ routine was applied followed by adjusting the threshold of the new image to define an edge. The FIJI Analyze Particles add-on was employed to automatically determine the area in pixels of each identified congruous particle. Application of ‘Find Particles’ first required setting the image threshold to define a particle edge in a binary image. One limitation of this procedure is that, to define a particle, the particle edge must be connected across the entire circumference of the particle and cannot be contiguous with the edge of another VLP. Consequently, the threshold was manually adjusted between 10 and 45 units to balance competing effects of closing the edges of as many individual VLPs as possible versus having the edges of multiple VLPs connect. The ‘Find Particles’ application provides a numbered contour map of particle outlines (Fig. 8C, F) with tabulated sizes of each identified particle.

### Gaussian mixture model (GMM) analysis

Gaussian mixture model analysis was performed in RStudio. The function to construct multi-normal quantile-quantile plots was written in house in RStudio. A single Gaussian distribution is defined by

1 $${\boldsymbol{p}}\left({\boldsymbol{x}}\right)=\frac{{\bf{1}}}{{\boldsymbol{\sigma }}\sqrt{{\bf{2}}{\boldsymbol{\pi }}}}{{\boldsymbol{e}}}^{-{\left({\boldsymbol{x}}-{\boldsymbol{\mu }}\right)}^{{\bf{2}}}/{\bf{2}}{{\boldsymbol{\sigma }}}^{{\bf{2}}}}$$

where determination of the two Gaussian parameters, µ and σ, are straight forward from the mean and standard deviation of the observed data, respectively. However, when the parent probability is a function of two or more different Gaussian distributions, the ability to accurately estimate true population parameters is much more difficult, even with the one-dimensional GMM data encountered in this study. For a one-dimensional GMM of *K* Gaussians,

2 $${\boldsymbol{p}}\left({\boldsymbol{x}}\right)=\mathop{\sum }\limits_{{\boldsymbol{k}}={\bf{1}}}^{{\boldsymbol{K}}}{{\boldsymbol{w}}}_{{\boldsymbol{k}}}{\boldsymbol{G}}\left({\boldsymbol{x}}{\rm{|}}{{\boldsymbol{\mu }}}_{{\boldsymbol{k}}},{{\boldsymbol{\sigma }}}_{{\boldsymbol{k}}}\right)$$

where G(x | µ_k_, σ_k_) is a Gaussian distribution defined by Eq. (1) for a set of parameters µ_k_ and σ_k_. The values ω_k_ is a collection of positive mixing coefficients, or weights assigned to each latent Gaussian distribution, under the constraint that

3 $$\mathop{\sum }\limits_{{\boldsymbol{k}}={\bf{1}}}^{{\boldsymbol{K}}}{{\boldsymbol{w}}}_{{\boldsymbol{k}}}={\bf{1}}$$

Thus, for an application with *K* intrinsic Gaussian distributions, 3*K*-1 parameters must be fit to the data. In context of measuring VLP area (or diameter) by AFM, the observed data has only one variable, VLP dimension. However, there are a presumed greater number of latent variables that define the intrinsic data structure, the underlying *K* Gaussian distributions. A robust alternative to non-linear least squares methods for fitting a GMM is estimating the latent parameters through a ML approach. ML methods optimize the likelihood of a set of parameters being correct, given a collection of observed data. For a GMM, the optimization criterion is expressed as

4a $${\boldsymbol{L}}\left({\boldsymbol{\theta }}{\rm{|}}{\boldsymbol{X}}\right)=\mathop{\prod }\limits_{{\boldsymbol{i}}={\bf{1}}}^{{\boldsymbol{N}}}\mathop{\sum }\limits_{{\boldsymbol{k}}-{\bf{1}}}^{{\boldsymbol{K}}}{{\boldsymbol{w}}}_{{\boldsymbol{k}}}{\boldsymbol{p}}\left({{\boldsymbol{x}}}_{{\boldsymbol{i}}}{\rm{|}}{{\boldsymbol{\theta }}}_{{\boldsymbol{k}}}\right)=\mathop{\prod }\limits_{{\boldsymbol{i}}={\bf{1}}}^{{\boldsymbol{N}}}\mathop{\sum }\limits_{{\boldsymbol{k}}-{\bf{1}}}^{{\boldsymbol{K}}}{{\boldsymbol{w}}}_{{\boldsymbol{k}}}{\boldsymbol{G}}\left({{\boldsymbol{x}}}_{{\boldsymbol{i}}},{{\boldsymbol{\mu }}}_{{\boldsymbol{k}}},{{\boldsymbol{\sigma }}}_{{\boldsymbol{k}}}\right)$$

or alternatively as the log likelihood

4b $${\boldsymbol{l}}\left({\boldsymbol{\theta }}\right)={\mathbf{log}}\,{\boldsymbol{L}}\left({\boldsymbol{\theta }}{\rm{|}}{\boldsymbol{X}}\right)=\mathop{\sum }\limits_{{\boldsymbol{i}}={\bf{1}}}^{{\boldsymbol{N}}}{\mathbf{log}}\,\left(\mathop{\sum }\limits_{{\boldsymbol{k}}-{\bf{1}}}^{{\boldsymbol{K}}}{{\boldsymbol{w}}}_{{\boldsymbol{k}}}{\boldsymbol{G}}\left({{\boldsymbol{x}}}_{{\boldsymbol{i}}},\,{{\boldsymbol{\mu }}}_{{\boldsymbol{k}}},{{\boldsymbol{\sigma }}}_{{\boldsymbol{k}}}\right)\right)$$

where the *N* observations are being described by a *K* factor GMM. The Expectation-Maximization (EM) algorithm is a robust and efficient method of optimizing Eqs. (4a) and (4b)^30,49^. The EM algorithm introduces a latent variable Z which is the collection of the *K* intrinsic Gaussian distributions in the GMM. Thus, the complete likelihood of the model is

5 $${\boldsymbol{L}}\left({\boldsymbol{\theta }}{\rm{|}}{\boldsymbol{X}},\,{\boldsymbol{Z}}\right)={\boldsymbol{p}}\left({\boldsymbol{X}},\,{\boldsymbol{Z}}{\rm{|}}{\boldsymbol{\theta }}\right)={\boldsymbol{p}}\left({\boldsymbol{Z}}{\rm{|}}{\boldsymbol{X}},{\boldsymbol{\theta }}\right)\,{\boldsymbol{p}}\left({\boldsymbol{X}}{\rm{|}}{\boldsymbol{\theta }}\right)$$

where the likelihood of the ϴ parameters, given a set of observations (X) and a presumed latent model (Z) is equal to the joint probability of observing X and Z given the GMM parameters ϴ. The EM model progresses in two main iterative steps. After an initial guess of ϴ, the Expectation Step estimates γ, the distribution of each component in Z given X and ϴ,

6 $${{\boldsymbol{\gamma }}}_{{{\boldsymbol{z}}}_{{\boldsymbol{i}}}={\boldsymbol{k}}}=\,{\boldsymbol{p}}\left({{\boldsymbol{Z}}}_{{\boldsymbol{i}}}={\boldsymbol{k}}\,{\rm{|}}\,{{\boldsymbol{X}}}_{{\boldsymbol{i}}}\right)=\,\frac{{\boldsymbol{p}}\left({{\boldsymbol{X}}}_{{\boldsymbol{i}}}\,{\rm{|}}\,{{\boldsymbol{Z}}}_{{\boldsymbol{i}}}={\boldsymbol{k}}\right){\boldsymbol{p}}\left({{\boldsymbol{Z}}}_{{\boldsymbol{i}}}={\boldsymbol{k}}\right)}{{\boldsymbol{p}}\left({{\boldsymbol{X}}}_{{\boldsymbol{i}}}\right)}\,=\,\frac{{{\boldsymbol{w}}}_{{\boldsymbol{k}}}{\boldsymbol{G}}\left({{\boldsymbol{x}}}_{{\boldsymbol{i}}},\,{{\boldsymbol{\mu }}}_{{\boldsymbol{k}}},{{\boldsymbol{\sigma }}}_{{\boldsymbol{k}}}\right)}{\mathop{\sum }\nolimits_{{\boldsymbol{k}}-{\bf{1}}}^{{\boldsymbol{K}}}{{\boldsymbol{w}}}_{{\boldsymbol{k}}}{\boldsymbol{G}}\left({{\boldsymbol{x}}}_{{\boldsymbol{i}}},\,{{\boldsymbol{\mu }}}_{{\boldsymbol{k}}},{{\boldsymbol{\sigma }}}_{{\boldsymbol{k}}}\right)}$$

where the numerator is the probability of X_i_ under component *k* and the denominator is the sum of probabilities across all components. The Maximization Step calculates new values of ϴ that maximizes the joint distribution of X and Z,

7 $${\hat{{\boldsymbol{\mu }}}}_{{\boldsymbol{k}}}=\frac{\mathop{\sum }\nolimits_{{\boldsymbol{i}}={\bf{1}}}^{{\boldsymbol{N}}}{{\boldsymbol{X}}}_{{\boldsymbol{i}}}{\boldsymbol{p}}\left({{\boldsymbol{Z}}}_{{\boldsymbol{i}}}={\boldsymbol{k}}\,{\rm{|}}\,{{\boldsymbol{X}}}_{{\boldsymbol{i}}},\,{{\boldsymbol{\theta }}}^{\left({\boldsymbol{t}}\right)}\right)}{\mathop{\sum }\nolimits_{{\boldsymbol{i}}={\bf{1}}}^{{\boldsymbol{N}}}{\boldsymbol{p}}\left({{\boldsymbol{Z}}}_{{\boldsymbol{i}}}={\boldsymbol{k}}\,{\rm{|}}\,{{\boldsymbol{X}}}_{{\boldsymbol{i}}},\,{{\boldsymbol{\theta }}}^{\left({\boldsymbol{t}}\right)}\right)}=\frac{\mathop{\sum }\nolimits_{{\boldsymbol{i}}={\bf{1}}}^{{\boldsymbol{N}}}{{\boldsymbol{X}}}_{{\boldsymbol{i}}}{{\boldsymbol{\gamma }}}_{{{\boldsymbol{Z}}}_{{\boldsymbol{i}}}={\boldsymbol{k}}}}{\mathop{\sum }\nolimits_{{\boldsymbol{i}}={\bf{1}}}^{{\boldsymbol{N}}}{{\boldsymbol{\gamma }}}_{{{\boldsymbol{Z}}}_{{\boldsymbol{i}}}={\boldsymbol{k}}}}$$

8 $$\widehat{{{\boldsymbol{\sigma }}}_{{\boldsymbol{k}}}^{{\bf{2}}}}=\,\frac{\mathop{\sum }\nolimits_{{\boldsymbol{i}}={\bf{1}}}^{{\boldsymbol{N}}}{{\boldsymbol{\gamma }}}_{{{\boldsymbol{Z}}}_{{\boldsymbol{i}}}={\boldsymbol{k}}}{\left({{\boldsymbol{X}}}_{{\boldsymbol{i}}}-{{\boldsymbol{\mu }}}_{{\boldsymbol{k}}}\right)}^{{\bf{2}}}}{\mathop{\sum }\nolimits_{{\boldsymbol{i}}={\bf{1}}}^{{\boldsymbol{N}}}{{\boldsymbol{\gamma }}}_{{{\boldsymbol{Z}}}_{{\boldsymbol{i}}}={\boldsymbol{k}}}}$$

9 $${\hat{{\boldsymbol{w}}}}_{{\boldsymbol{k}}}=\,\frac{\mathop{\sum }\nolimits_{{\boldsymbol{i}}={\bf{1}}}^{{\boldsymbol{N}}}{{\boldsymbol{\gamma }}}_{{{\boldsymbol{Z}}}_{{\boldsymbol{i}}}={\boldsymbol{k}}}}{{\boldsymbol{N}}}$$

where ϴ^(t)^ is the previous estimate of ϴ. The algorithm iterates between the E-Step and M-Step until a convergence criterion, such as the log-likelihood (Eq. (4b) agreeing to 6 decimal places in subsequent iterations or a maximum number of allowed iterations is reached.

### Expectation-maximization (EM) algorithm

All analyses were performed using R^50^ in RStudio^51^; the package ‘mixtools’^52^ was employed to apply a GMM to the VLP areas or widths extracted from the AFM images. Convergence of the EM algorithm was capped at 1000 iterations. A function to construct multi-normal quantile-quantile plots was written in house. A regression function that relates the observed data and theoretical quantiles to statistically validate the GMM goodness of fit was written in house.

### Conclusions

Gaussian Mixture Models (GMMs), optimized by the Expectation-Maximization (EM) algorithm, is a beneficial and effective tool for analyzing AFM images of mixed-size nanoparticles, in this case virus-like particles (VLPs). Univariate statistics that assume a single normal distribution of particle sizes (either area or width) do not convey the nuances of multiple nanoparticle populations within a single image or a collection of images. The GMM, driven by the EM algorithm, can rapidly and robustly extract the parameters of heterogeneous nanoparticle sub-populations. However, these methods are only effective if the sub-populations conform to the assumed Gaussian distribution. Analyses of VLPs by compressing the nanoparticles with a rigid AFM tip during imaging is an intriguing option to implicitly extract information about potential internal VLP changes. The theory, espoused previously^27^, postulates that the compressibility of the VLP may indicate internal changes prior to those observed externally. This work builds on these observations and hints that VLP morphological changes may potentially occur in more discrete stages and less as a homogeneous continuum. This suggestion is supported by the existence of discrete sub-populations of VLP sizes elucidated by the GMM. Were the VLP changes continuous, as reflected in the VLP compression, a single distribution of VLP sizes would be observed by the GMM.

### Reporting summary

Further information on research design is available in the Nature Research Reporting Summary linked to this article.

### Supplementary information

REPORTING SUMMARY

## Data Availability

The datasets used and/or analyzed during the current study are available from the corresponding authors on reasonable request.

## Code availability

The code used and/or analyzed during the current study are available from the corresponding authors on reasonable request.
